# Intra‐operative oxygen concentration could affect postoperative morbidity—results of a randomised controlled feasibility trial (PULSE Ox)

**DOI:** 10.1002/anr3.70109

**Published:** 2026-10-19

**Authors:** A. F. Cumpstey, J. Stygall, A. D. Clark, M. Minnion, H. Moyses, D. S. Martin, M. R. Edwards, M. Feelisch, M. P.W. Grocott

**Affiliations:** ^1^ Peri‐operative and Critical Care Theme, NIHR Southampton Biomedical Research Centre University Hospital Southampton Southampton UK; ^2^ Clinical & Experimental Sciences, Faculty of Medicine University of Southampton Southampton UK; ^3^ Peninsula Medical School University of Plymouth Plymouth UK

**Keywords:** cancer, hyperoxia, postoperative cognitive dysfunction, postoperative outcomes, pulmonary complications

## Abstract

The World Health Organisation recommends that all anaesthetised patients receive F_I_O_2_ 0.8 during surgery to reduce the risk of surgical site infections but did not consider the effect this might have on other clinical outcomes. A Cochrane systematic review concluded that insufficient evidence exists for routinely administering high oxygen concentrations intra‐operatively to reduce surgical site infections, and that doing so might increase mortality. A feasibility trial was conducted looking at the feasibility of controlling intra‐operative F_I_O_2_ and measuring systemic redox state. Here we explore further clinical outcomes from this study regarding postoperative complications and cognitive recovery. Twenty‐eight adult patients undergoing major abdominal surgery for cancer resection were randomly allocated to receive either conservative (F_I_O_2_ = 0.3, n = 8), typical (F_I_O_2_ = 0.55, n = 10) or recommended (F_I_O_2_ = 0.8, n = 10) oxygen throughout anaesthesia. Rates of radiologically reported atelectasis, cognitive recovery and infective postoperative morbidity were all collected up to 7 days after surgery. Total intensive care unit length of stay was also recorded. Higher F_I_O_2_ levels were associated with lower rates of atelectasis ‐ 6 (75%) versus 8 (80%) versus 3 (30%) for F_I_O_2_ = 0.30 versus 0.55 versus 0.80, respectively; p = 0.045. Postoperative cognitive recovery scores (0.7 (3.1) versus −0.1 (2.5) versus −1.8 (3.3), p = 0.277), Postoperative Morbidity Survey infection scores (5 (71%) versus 5 (56%) versus 3 (33%), p = 0.307) and intensive care unit length of stay (3 (2–4) versus 2.5 (2.00–3.75) versus 3 (2.00–5.25) days, p = 0.870) did not differ between groups. Changing the administered intra‐operative oxygen concentration may alter postoperative clinical outcomes, and adequately powered clinical studies are urgently needed to investigate the impact of this.

## Introduction

Hyperoxia has been associated with harm in acute illness [1]. However, in other clinical settings, such as intensive care populations, it is often not possible to draw clear conclusions about the effects of higher or lower oxygenation strategies on specific outcomes, such as lung injury, myocardial infarction or stroke [2]. In surgery, despite being a ubiquitous part of almost every general anaesthetic procedure, knowledge about how the use of supplemental oxygen affects postoperative outcomes remains remarkably limited [3].

Most previous peri‐operative oxygen research has focussed primarily on investigating whether higher intra‐operative oxygen concentrations might improve surgical site infections (SSI), but findings remain controversial [4]. The World Health Organisation (WHO) recommends that all anaesthetised patients receive F_I_O_2_ = 0.8 during surgery to reduce the risk of SSI but did not consider the effect this might have on other clinical outcomes [5, 6, 7]. Cochrane concluded insufficient evidence exists for routinely administering high oxygen concentrations intra‐operatively to reduce SSI, and that doing so might increase mortality [8].

Most peri‐operative mortality and morbidity continue to occur during the postoperative period secondary to surgical complications [9]. In the UK, around 16% of surgical patients experience complications, tripling the cost of admission for this group [10]. A recent observational study concluded that administration of supraphysiological oxygen levels during surgery was associated with a higher incidence of kidney, myocardial and lung injury measured biochemically [11]. However, relatively little is known about whether changing intra‐operative oxygen concentration during non‐cardiac surgery affects rates of other postoperative complications that are important to patients, such as functional and cognitive recovery. The mechanism for any effect oxygen might have on clinical outcomes after surgery has also been questioned [12].

We hypothesised that exposure to hyperoxia would elicit adaptive metabolic changes to maintain redox balance that might then lead to changes in postoperative complications and a feasibility trial was conducted. The feasibility outcomes and how hyperoxia altered systemic redox state in these patients have already been reported separately [13]. The potential impact of changing F_I_O_2_ on postoperative complications, including pulmonary complications and cognitive recovery, is explored here. This paper reports the clinical outcomes collected in the feasibility trial.

## Methods

### Study design and participants

This single‐centre, prospective, interventional, blinded randomised trial was conducted at University Hospital Southampton between 2018 and 2021. The trial was sponsored by the University Hospital Southampton NHS Foundation Trust and approved by the South Central‐Hampshire A Research and Ethics Committee (reference 18/SC/0018). It was prospectively registered on Clinicaltrials.gov (NCT03552627) on 01/03/2018.

Adults undergoing major abdominal surgery and needing an arterial and central venous catheter as part of their anaesthetic management were eligible. Patients were not eligible to participate if they were pregnant; undergoing neurosurgery, cardiac surgery or needing one‐lung ventilation at any point during their procedure; had baseline peripheral oxygen saturation (SpO_2_) <90%; had severe COPD (defined as Global Initiative for Chronic Obstructive Lung Disease stage 3 or 4), interstitial lung disease or another inflammatory lung condition, including being on home oxygen; had a BMI >35 kg.m^−2^ or any documented concerns about a potentially difficult airway; or had sickle cell disease or thalassaemia. An amendment (notified 12/12/2019, approved 20/12/2019) added unsuitability for total intravenous anaesthesia or for fixed PEEP as additional exclusion criteria. All participants provided written informed consent.

### Interventions

Participants received F_I_O_2_ = 0.8 (WHO recommendation), F_I_O_2_ = 0.55 (typical practice) or F_I_O_2_ = 0.3 oxygen (conservative administration) at random (1:1:1 allocation) throughout their anaesthesia, from the start of their pre‐oxygenation period through to the point of leaving the operating theatre. Anaesthesia was maintained using a total intravenous anaesthetic technique (target‐controlled infusions of propofol and remifentanil) with rocuronium for neuromuscular blockade. A pressure control mode of ventilation delivered tidal volumes between 6 and 8 ml.kg^−1^ (ideal body weight) with a PEEP of 5 cmH_2_O. No patients received dexamethasone or other steroids, but ondansetron was given for antiemesis. Other aspects of anaesthetic management were left to the discretion of the treating anaesthetist.

### Outcomes

Mortality was recorded at 30 days. Length of stay (LOS) was collected on discharge from the ICU and/or from hospital. Postoperative morbidity was assessed on day 7 using the Postoperative Morbidity Survey (POMS), a 9‐point survey which screens for postoperative complications in different organ systems and has been validated for use up to 15 days after surgery [14, 15]. Postoperative pulmonary complications were defined using the Standardised Endpoints in Peri‐operative Medicine ‐ Core Outcome Measures for Peri‐operative and Anaesthetic Care (Step‐COMPAC) outcome definitions [16]. Atelectasis was recorded if atelectasis was independently reported by a qualified reporting clinician on any routinely performed postoperative radiological chest imaging within 7 days of surgery. Full blood count, urea, creatinine, liver function tests (alkaline phosphatase, ALP; alanine aminotransferase, ALT), C‐reactive peptide (CRP) and troponin were measured using automated chemical analysers before surgery, at the end of surgery and 7 days after surgery.

### Postoperative cognitive recovery

A battery of six neuropsychological tests was used to compare cognitive scores obtained pre‐operatively with performance 7 days postoperatively. These commonly used tests were selected to assess cognitive function under broad‐based categories of memory, attention and concentration, perceptuomotor ability and verbal fluency. Where available, parallel forms were employed to limit learning effects between assessments. All assessments were conducted by the same trained clinician. The assessments were:

1. Orientation screening: all participants were asked to give their name, date of birth and current location to screen for significant cognitive impairment. If any participant was unable to answer these questions the assessment was terminated.
2. Digit Span Forwards and Digit Span Backwards: these tests from the Wechsler Adult Intelligence Scale‐Revised (WAIS‐R) manual were used to measure working memory and attention [17]. Both tasks consist of several sequences of numbers, read out aloud by the examiner at a rate of one digit per second and increasing in length by one number each time until the participant fails to repeat the sequence (forwards or backwards, as appropriate) correctly.
3. Word List Learning Test: a word list adapted from the widely used Rey Auditory Verbal Learning Test assessed short‐term memory [18]. Participants were asked to recall as many words as they could from the verbal presentation of a 15‐word list and measure short‐term verbal memory.
4. Trail Making Test Part A and Part B: these tests, which originally formed part of the Army Individual Test Battery, assessed motor speed, attention and mental flexibility [19]. Participants were asked to draw a continuous line as quickly as possible through circles containing either consecutive numbers or ascending numbers and letters. Time to completion was recorded.
5. The Controlled Oral Word Association (COWA) task: this test measured verbal fluency [20, 21]. The participants were asked to say as many words as they could think of that began with a specific letter of the alphabet, excluding proper nouns, numbers and the same word with different suffixes.

To make optimal use of the cognition data, each participant's pre‐ and postoperative scores were compared to create a change score. Standardised change scores were calculated by subtracting the pre‐operative score from the postoperative score and then dividing this change in score by the pre‐operative standard deviation for each individual test. Where improvement in performance in any test was reflected by a lower score (the timed trail making tests), the direction of the score was reversed by multiplying by −1 so that all improvements in performance gave rise to a positive change score, and any deterioration to a negative score. A global z change score was obtained for each participant by summing the change scores of the individual tests. When a participant was unable to complete a task because of cognitive difficulties, a score of two standard deviations below the mean for the whole group was recorded for that test.

### Mood assessment

It has been suggested that mood may impact upon neuropsychological performance [22]. Therefore, the state subscale of the Spielberger State–Trait Anxiety Inventory (STAI) and the Centre for Epidemiological Studies Depression Scale (CES‐D) were administered at each assessment [23, 24]. Fatigue was also assessed using the Fatigue Severity Scale [25].

### Randomisation and blinding

Each participant was randomly assigned a F_I_O_2_ using predetermined sealed envelopes generated by an independent third party. All participants, research team members and the Sponsor's study team remained masked to the allocated intervention throughout the trial. All anaesthetic oxygen monitoring was kept covered during the operative period, keeping research team members blinded to the allocation; however, the treating clinical anaesthetist (not blinded) was able to check under these to safely deliver the allocated F_I_O_2_. Electronic recordings of the anaesthetic delivery, collected contemporaneously during each individual procedure, allowed intra‐operative administration to be unblinded at the end of the study after all other analysis had been completed.

### Sample size and statistical analyses

A sample size of 39 participants was determined for the feasibility trial based on redox markers assessed biochemically, as described previously [13]. As the clinical outcomes reported here were all either secondary or exploratory outcomes, the study was not powered for assessing these. An intention‐to‐treat statistical analysis was performed. Results are presented as mean (SD) or median (IQR) depending on normality testing (Shapiro–Wilk) and chi‐squared, ANOVA or Kruskall–Wallis statistical tests were used as appropriate. All statistics were calculated using GraphPad Prism or STATA. No interim analyses were performed. Statistical significance was assumed at p = 0.05.

## Results

Between 27/09/2018 and 08/05/2021, 28 patients completed the study before the trial had to stop early due to the COVID‐19 pandemic. A CONSORT diagram of the trial has been reported previously [13]. Baseline characteristics are reported in Table 1.

**Table 1:** Baseline characteristics of participants in PULSE Ox by interventional trial group. Values are presented as mean (SD) or n [%].

Baseline characteristics	F_I_O_2_ 0.3	F_I_O_2_ 0.55	F_I_O_2_ 0.8
	(n = 8)	(n = 10)	(n = 10)
Age, years	64.5 (4.7)	67.3 (3.2)	61.3 (2.9)
Sex			
Male	5 [62.5%]	3 [30%]	5 [50%]
Ethnicity			
White British	8 [100%]	10 [100%]	10 [100%]
Other	0 [0%]	0 [0%]	0 [0%]
Weight, kg	77.7 (16.1)	79.1 (10.5)	73.9 (16.7)
BMI, kg.m^−2^	27.3 (4.8)	28.6 (3.5)	25.7 (4.8)
Current/ex‐smoker	5 [63%]	6 [60%]	7 [70%]
Pre‐operative observations			
SpO_2_, %	97.3 (1.5)	97.1 (1.3)	97.7 (1.2)
Heart rate, beats.min^−1^	81.8 (10.2)	79.2 (12.2)	76.7 (20.3)
Systolic blood pressure, mmHg	150.4 (18.4)	142.6 (15.2)	135.2 (14.6)
Diastolic blood pressure, mmHg	88.0 (11.3)	86.1 (6.9)	80.8 (11.5)
Temperature, °C	36.3 (0.3)	36.5 (0.4)	36.7 (0.3)
P‐POSSUM scores			
Physiology	16.1 (2.6)	15.7 (2.1)	15.2 (2.8)
Operative	22.5 (8.6)	21.4 (6.6)	22.8 (5.1)
Mortality	24.7 (32.7)	13.9 (27.4)	17.3 (27.3)
Morbidity	49.9 (35.7)	54.6 (26.5)	55.9 (30.6)
Site of primary malignancy			
Hepatobiliary	2 [25%]	5 [50%]	3 [30%]
Colorectal	4 [50%]	4 [40%]	5 [50%]
Bladder	1 [12.5%]	0 [0%]	1 [10%]
Other (cervical/neuro‐endocrine)	1 [12.5%]	1 [10%]	1 [10%]
Cardiovascular disease	2 [25%]	1 [10%]	2 [20%]
Hypertension	2 [25%]	2 [20%]	2 [20%]
Respiratory disease	1 [12.5%]	3 [30%]	0 [0%]
Type 2 diabetes	1 [12.5%]	1 [01%]	2 [20%]
Kidney disease	1 [12.5%]	1 [10%]	2 [20%]

At 30 days, all participants were alive, one patient in the F_I_O_2_ = 0.3 group had been readmitted to hospital and three other patients had not yet been discharged (one in the F_I_O_2_ = 0.3 group and two in the F_I_O_2_ = 0.8 group). There was no difference in intensive care LOS (median (min ‐ max) 3 (2–6) versus 2.5 (1–7) versus 3 (0–7) days for F_I_O_2_ = 0.30 versus 0.55 versus 0.80, respectively; p = 0.8699) or overall postoperative LOS (11 (5–35) versus 11.5 (5–23) versus 13.5 (4–41) with F_I_O_2_ = 0.30 versus 0.55 versus 0.80, respectively; p = 0.9101), see Figure S1. Day 7 Postoperative Morbidity Survey (POMS) scores are shown in Table S1.

Half of all patients continued antibiotics at day 7. Patients in the F_I_O_2_ = 0.3 group had the highest rate of infection‐related morbidity (n = 5, 71.4%), while the patients in the F_I_O_2_ = 0.8 group had the lowest rate of infection‐related morbidity (n = 3, 33.3%) assessed by POMS. However, this difference was not statistically significant, p = 0.307 (Table S1).

Other infections (not meeting POMS criteria) were recorded in three (37.5%) patients in the F_I_O_2_ = 0.30 group, three (30%) patients in the F_I_O_2_ = 0.55 group and no patients in the F_I_O_2_ = 0.80 (p = 0.371). No wound or urinary tract infections were recorded. Two patients were treated for abdominal collections, one in the F_I_O_2_ = 0.30 group and one in the F_I_O_2_ = 0.55 group. Three patients required treatment for postoperative pneumonia; two in the F_I_O_2_ = 0.30 and one in the F_I_O_2_ = 0.55 group. Overall, the rate of postoperative pneumonia did not differ between the three groups (p = 0.233). There was no difference in time to recommencing enteral nutrition between the groups (5.4 (3.0) versus 5.0 (3.8) versus 4.9 (3.6) with F_I_O_2_ = 0.30 versus 0.55 versus 0.80, respectively; p = 0.952).

### Postoperative pulmonary complications

No episodes of acute respiratory distress syndrome (ARDS) or pulmonary aspiration were recorded. There was no difference in the number of participants continuing mechanical ventilation at the end of surgery (p = 0.183) (Figure S2A). The tracheas of all but two of the patients who continued mechanical ventilation were extubated the day after surgery, and everyone's tracheas were extubated within 3 days of surgery ending (Figure S2C). Significantly lower rates of atelectasis were seen in the group receiving F_I_O_2_ = 0.8 (p = 0.045) (Figure S2B). Twenty‐seven participants (96%) continued supplemental oxygen for at least 1 day after surgery but there was no difference between the three interventional groups in the duration of supplementary postoperative oxygen administration (p = 0.112) (Figure S2D).

### Pathology laboratory measurements

Mean ALT concentrations were higher in the F_I_O_2_ = 0.3 group at the end of surgery compared to both the F_I_O_2_ = 0.55 and the F_I_O_2_ = 0.8 groups. However, one patient in the F_I_O_2_ = 0.3 group who was having surgery to remove a bile duct cholangiocarcinoma had an ALT of 595 IU.l^−1^ at the end of surgery (400 IU.l^−1^ higher than any other participant in any group). Baseline CRP was also higher in this patient, explaining why mean CRP was higher at baseline in the F_I_O_2_ = 0.3 group even though CRP did not change between intervention groups overall. Another patient in the F_I_O_2_ = 0.3 group had a troponin concentration of 110 ng.ml^−1^ at the end of surgery (approximately 50 ng.ml^−1^ higher than the next highest recorded value in the study), which had returned to baseline levels by day 7 (Table S2).

### Postoperative cognitive recovery

All 28 participants completed the cognitive assessment panel before their operation. Four patients were discharged before completing the postoperative assessment on day 7 (two in the F_I_O_2_ = 0.3 group and one from each of the F_I_O_2_ = 0.55 and F_I_O_2_ = 0.8 groups) (Table S3).

Fatigue scores significantly increased following surgery in all groups (p = 0.0001), but there was no difference in fatigue scores between interventional groups. Mean (SD) scores were 32.0 (12.7), 33.1 (12.2) and 27.5 (7.8) with F_I_O_2_ = 0.30, 0.55 and 0.80, respectively pre‐operatively and 46.0 (16.7), 45.9 (9.9) and 43.0 (10.6), respectively postoperatively (Figure 1A).

**Figure 1:**
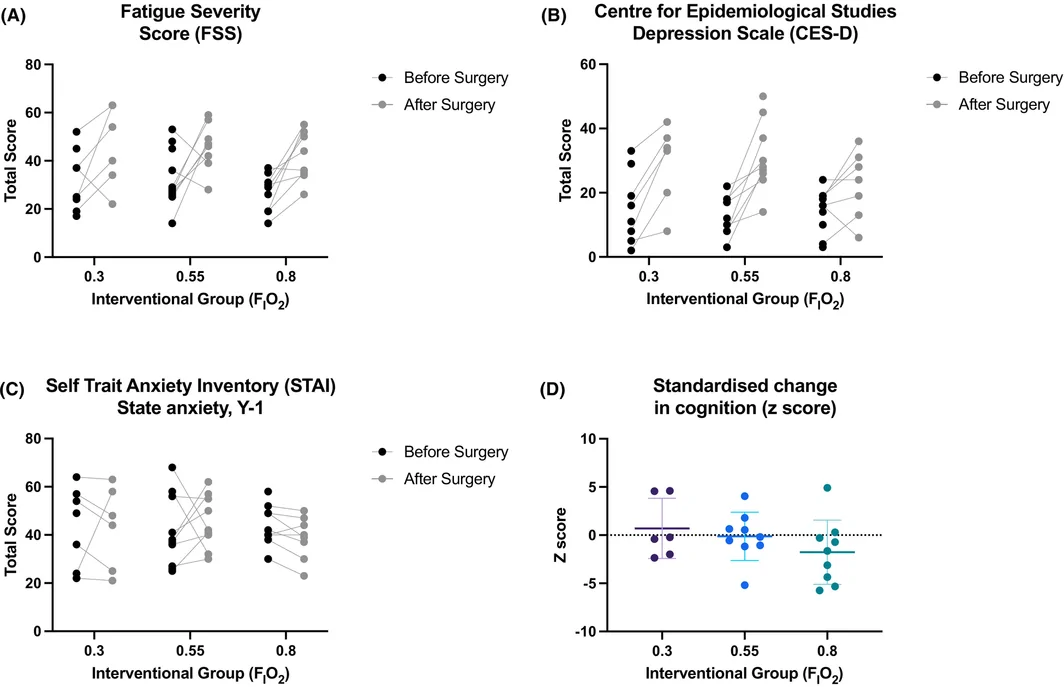
(A) Self‐assessment scores for fatigue; (B) depression; (C) anxiety; (D) overall standardised z scores for cognitive recovery after surgery.

Depression scores, using the Centre for Epidemiological Studies Depression Scale (CES‐D), also significantly increased following surgery in all groups (p = 0.0001), with no difference between interventional groups. Mean (SD) scores were 15.4 (11.1), 12.8 (6.1) and 14.3 (6.8) with F_I_O_2_ = 0.30, 0.55 and 0.80, respectively pre‐operatively and 29.0 (12.6), 31.2 (11.1) and 22.4 (10.5), respectively postoperatively (Figure 1B).

Anxiety scores, using the Spielberger State–Trait Anxiety Inventory (STAI), neither changed significantly following surgery (p = 0.902) nor between oxygen groups (p = 0.941). Mean (SD) scores were 41.0 (6.1), 41.2 (4.7) and 42.8 (2.9) with F_I_O_2_ = 0.30, 0.55 and 0.80, respectively pre‐operatively and 43.2 (7.0), 45.4 (3.7) and 38.8 (3.1), respectively postoperatively (Figure 1C).

Global standardised change (z) scores were highest (biggest improvement in cognitive performance after surgery) in the F_I_O_2_ = 0.3 group and lowest (biggest deterioration in cognitive performance after surgery) in the F_I_O_2_ = 0.8 group, although these differences did not reach statistical significance (p = 0.277). Mean (SD) values were + 0.70 (3.12) with F_I_O_2_ = 0.30, −0.13 (2.50) with F_I_O_2_ = 0.55 and −1.78 (3.34) with F_I_O_2_ = 0.80 (Figure 1D).

## Discussion

This trial of 28 patients undergoing major non‐cardiac surgery for cancer resection explored whether different intra‐operative oxygen concentrations during anaesthesia may translate into different postoperative outcomes. We successfully captured and reported pulmonary complications, postoperative morbidity scores, postoperative cognitive recovery, readouts of tissue damage and other clinical biomarkers. Administration of F_I_O_2_ = 0.8 throughout surgery was associated with lower rates of atelectasis, but no significant differences were seen in rates of other pulmonary complications, length of stay in the ICU, mortality or postoperative cognitive recovery. However, these findings should be considered exploratory in nature and merit further investigation.

Identifying the optimal F_I_O_2_ to administer peri‐operatively remains an important international research priority. Current guidelines recommending administration of F_I_O_2_ = 0.8 throughout surgery have been widely challenged by anaesthetists and do not appear to be routinely followed by many clinicians internationally [26, 27, 28]. Although many previous trials have compared the administration of ‘high’ (typically F_I_O_2_ = 0.8) versus ‘low’ (usually F_I_O_2_ = 0.3 or similar) oxygen during anaesthesia, these have almost universally focused on the effects on SSI [3]. Evidence for how peri‐operative oxygen administration alters other peri‐operative outcomes remains remarkably limited.

PULSE Ox was not designed or powered to be a full clinical effectiveness study ‐ it was powered on mechanistic outcomes and only recruited 28 patients in total [13]. If oxygen indeed affected peri‐operative mortality, many thousands of patients would be needed to securely identify this. In the largest intra‐operative oxygen trial to date, recruiting 5749 patients in total, only 30 patients across both groups died in hospital [29]. However, patients continue to prioritise outcomes other than mortality, including the quality of their functional and cognitive recovery after surgery [30]. In this context, the outcomes of PULSE Ox are hypothesis generating to support the design of larger clinical trials that are urgently needed.

Although in this study, the incidence of atelectasis was lowest in the F_I_O_2_ = 0.8 group, this is unexpected and contrasts with most, but not all, previous evidence and a type 1 error is possible given the small sample size [4]. Two large retrospective observational studies have previously reported separately that exposure to higher intra‐operative oxygen concentrations is associated with increased rates of major respiratory complications after surgery although atelectasis was not reported specifically in either case [11, 31]. However, a large randomised controlled trial (PROXI) found no difference in rates of pneumonia or atelectasis in 1400 patients exposed to either F_I_O_2_ = 0.3 or 0.8 [32]. Given the smaller sample size in this study, the observed association may be confounded by small differences within the groups. For example, ventilation was continued postsurgery in more patients in the F_I_O_2_ = 0.8 group. Longer ventilation (with PEEP and pressure‐controlled tidal volumes) may have helped prevent atelectasis in the immediate postoperative period. It might also be due to methodological differences, particularly around the start of anaesthesia. No previous trial has included the pre‐oxygenation period within the oxygen intervention, meaning that, for the first time in this study, no patients, including those in the F_I_O_2_ = 0.8 group, were exposed to F_I_O_2_ = 1.0, which would only be expected to increase absorption atelectasis, at any point.

The reduction observed in overall cognitive recovery scores merits further investigation in larger cohorts. Other data exploring how intra‐operative oxygen affects cognitive outcomes from non‐cardiac surgery is scarce. However, cerebral oxidative stress has been implicated in the development of cognitive impairment and other neurodegenerative diseases, and neurons are particularly susceptible to free radical damage because of their high adenosine triphosphate requirements [33, 34, 35]. Systemic inflammation, particularly neuro‐inflammation, is thought to impair neurological function specifically after surgery and may lead to postoperative cognitive decline, but the role oxygen might or might not play in this has not been widely studied [36]. A recent systematic review for possible risk factors for delirium or cognitive decline following coronary artery bypass grafting surgery does not even list or identify oxygen concentration as a possible intra‐operative risk factor [37]. Decreased oxygen content after cardiopulmonary bypass has been associated with greater postoperative cognitive decline in cardiac surgical patients, but as total oxygen content is dependent on both haemoglobin concentration and oxygen saturation, it is difficult to separate the effects of both surgical blood loss and intra‐operative oxygen administration [38]. In another trial, no association was identified between arterial hyperoxia during cardiopulmonary bypass and postoperative cognitive decline following bypass grafting procedures in over 1000 patients, but synchronous intra‐operative hypothermia may have helped protect against this and was not otherwise controlled for in that study [39]. However, a smaller pilot study (n = 93) reported that both peak and mean PaO_2_ values were higher in elderly patients who developed postoperative delirium after cardiac surgery compared to those who did not, supporting the findings of this study [40].

Our study had many strengths and several limitations. One important methodological strength was the successful intervention concealment, which has rarely been achieved in clinical oxygen trials. Another strength was including the pre‐oxygenation and emergence periods within the randomised intervention, a feature unique to this study. Adherence to the randomisation intervention was remarkably good, leading to the mean oxygen concentration being very close to target oxygen concentrations in every group. However, an important limitation is that every patient in PULSE Ox underwent a long procedure to treat a primary malignancy and because of the complexity of the surgical procedures performed and the likely length of time needed to recover and benefit from them, most patients had relatively few baseline co‐morbidities. All patients were White British. This limits the applicability of the results described to other peri‐operative settings. The study was not powered for clinical outcomes and had to stop recruitment early because of the COVID‐19 pandemic, limiting its power further. With relatively small numbers in each group, it cannot be excluded that other unmeasured confounders might have affected the reported results despite adequate randomisation. As the reported clinical outcomes were all exploratory outcomes, no formal correction for multiple comparison was performed, increasing the possibility of type 1 errors. The results should therefore be treated as exploratory. The interval between study completion and publication is also acknowledged. During this period, analyses and dissemination of the feasibility study outcomes were completed, and these results informed the design of a planned clinical effectiveness trial.

In conclusion, administering different intra‐operative oxygenation concentrations during major surgery may alter postoperative clinical outcomes important to patients, clinicians and policymakers. Adequately powered clinical studies are urgently needed to determine the optimal approach to peri‐operative oxygenation to minimise overall harm and not limited to reducing surgical site infections.

## Supporting information


**Table S1.** Postoperative Morbidity Survey (POMS) results by interventional group. Results are presented as the number of patients and the percentage of patients in that interventional group. Percentages for each individual POMS are calculated from the total number of patients who were still in hospital at the time of assessment (with or without POMS). Percentages do not total 100% as often more than one organ system was affected simultaneously in an individual patient with POMS. P‐values calculated using Chi‐squared test (no calculation possible where POMS score = 0. a = waiting transport, b = raised inflammatory markers and c = further stoma teaching required).
**Table S2.** Pathology laboratory values for PULSE Ox participants immediately before surgery, at the end of surgery and 7 days after surgery according to interventional F_I_O_2_. All values are expressed as either mean (SD) or median [IQR], as appropriate. P‐values are calculated for differences in the change scores (postsurgery value ‐ presurgery value) between the three interventional groups using either one‐way ANOVA or Kruskal–Wallis, as appropriate.
**Table S3.** Descriptive statistics for postoperative cognitive recovery tests by question and by interventional group, both pre‐operatively and 7 days after surgery.
**Figure S1.** Length of stay (LOS) by interventional group. Plot A shows intensive care LOS and Plot B shows total postoperative LOS. Error bars represent the median and IQR.
**Figure S2.** Postoperative respiratory complications by interventional group. Panel A shows the number of patients who continued mechanical ventilation (MV) at the end of surgery. Panel B shows rates of atelectasis post operatively. Panel C shows the duration in days that mechanical ventilation continued after the end of surgery. Panel D depicts the number of days within the first week after surgery that the patients received further supplemental oxygen.
