# Supplementary material for: Intra‐operative oxygen concentration could affect postoperative morbidity—results of a randomised controlled feasibility trial (PULSE Ox)
Source: Anaesth Rep. 2026 Oct 19;14(2):e70109. doi: 10.1002/anr3.70109 (PMC13680861; doi:10.1002/anr3.70109)
Supplement: Supplementary file 1 [file ANR3-14-e70109-s001.docx]

**Supplementary tables and figures for: Intra-operative oxygen concentration could affect postoperative morbidity – results of a randomised controlled feasibility trial (PULSE Ox)**

**Table S1:** Postoperative Morbidity Survey (POMS) results by interventional group. Results are presented as the number of patients and the percentage of patients in that interventional group. Percentages for each individual POMS are calculated from the total number of patients who were still in hospital at the time of assessment (with or without POMS). Percentages do not total 100% as often more than one organ system was affected simultaneously in an individual patient with POMS. P-values calculated using Chi-squared test (no calculation possible where POMS score = 0. a = waiting transport, b = raised inflammatory markers and c = further stoma teaching required).

**Table S2:** Pathology laboratory values for PULSE Ox participants immediately before surgery, at the end of surgery and 7 days after surgery according to interventional F_I_O_2_. All values are expressed as either mean (SD) or median [IQR], as appropriate. P-values are calculated for differences in the change scores (postsurgery value - presurgery value) between the three interventional groups using either one-way ANOVA or Kruskal-Wallis, as appropriate.

**Table S3:** Descriptive statistics for postoperative cognitive recovery tests by question and by interventional group, both pre-operatively and 7 days after surgery.

**Figure S1:** Length of stay (LOS) by interventional group. Plot A shows intensive care LOS and Plot B shows total postoperative LOS. Error bars represent median and IQR.

Figure S2: Postoperative respiratory complications by interventional group. Panel A shows the number of patients who continued mechanical ventilation (MV) at the end of surgery. Panel B shows rates of atelectasis post operatively. Panel C shows the duration in days that mechanical ventilation continued after the end of surgery. Panel D depicts the number of days within the first week after surgery that the patients received further supplemental oxygen.
